# Peri‐Implant Soft Tissue Increase at Small Buccal Bone Dehiscences With Either Volume‐Stable Collagen Matrix or Connective Tissue Graft: A Randomized Controlled Trial

**DOI:** 10.1111/clr.14430

**Published:** 2025-03-19

**Authors:** Francesco Ferrarotti, Giacomo Baima, Giulia Mohammadi, Clelia Carboncini, Federica Romano, Mario Aimetti

**Affiliations:** ^1^ Department of Surgical Sciences C.I.R. Dental School, University of Turin Turin Italy

**Keywords:** collagen matrix, connective tissue graft, dental implants, esthetics, patient‐reported outcomes, soft tissue augmentation

## Abstract

**Objectives:**

This randomized clinical study compared the profilometric measurements of the buccal tissue volume at sites augmented using a volume‐stable collagen matrix (VCMX) or connective tissue graft (CTG) simultaneously to implant placement in the presence of small buccal bone dehiscence (SBBD ≤ 3 mm).

**Methods:**

Forty‐four patients with SBBD were treated with soft tissue augmentation (STA) simultaneous to implant placement using VCMX or SCTG. Clinical measurements and 3D intraoral scans were collected prior to STA (BL), at 1, 3 months, and 1 year after prosthetic loading. Digital files were superimposed to compare profilometric volume on the buccal profile (primary outcome); peri‐implant health, radiographic bone levels, and patient‐reported outcome measures (PROMs) were also assessed.

**Results:**

Both treatments achieved a significant STA at 3 months, with a slight decrease observed from 1 month. At 3 months, the mean increase was 1.07 mm (SD 0.22) for VCMX and 1.22 mm (SD 0.44) for the CTG group (*p* = 0.156). PROMs revealed a difference in the perception of the bleeding at day 1, pain at 2 and 3 days, and swelling at 3 days favoring VCMX (*p* < 0.05). At 1 year, no intergroup difference in probing pocket depth, bleeding, and recession was detected, but CTG provided higher stability than VCMX in terms of profilometric measurements (0.21 mm [SD 0.32] vs. −0.05 mm [SD 0.36], respectively; *p* = 0.014) and radiographic bone levels (0.09 mm [SD 0.65] vs. −0.34 mm [SD 0.70]; *p* = 0.038).

**Conclusion:**

For implant placement at posterior sites with small buccal bone dehiscences, CTG and VCMX resulted in an initially comparable volume augmentation and clinical parameters, with VCMX leading to better PROMs. At 1 year, CTG maintained slightly higher profilometric stability and bone levels.

**Trial Registration:**

ClinicalTrials.gov identifier: NCT05466006 (https://classic.clinicaltrials.gov/ct2/show/NCT05466006)

## 1 Introduction

Alveolar ridge changes after tooth extraction induce volume deficiencies both at hard and soft tissue levels, negatively affecting the outcomes of subsequent implant‐supported restorations (Araújo and Lindhe 2009; Cardaropoli et al. 2003; Seibert 1983; Tan et al. 2012). In this context, guided bone regeneration (GBR) is indicated in the presence of severe ridge defects or major bone dehiscences after implant insertion (Jensen et al. 2023). In the presence of a minor horizontal defect or small buccal bone dehiscences (SBBD; < 3 mm from the intraosseous portion of the implant), soft tissue augmentation (STA) has been considered a viable approach to optimize the predictability of the outcomes of implant therapy (Stefanini et al. 2023; Thoma et al. 2019; Zuercher et al. 2023).

STA aims at changing the soft tissue phenotype around dental implants in terms of soft tissue thickness (STT) and/or keratinized mucosa width (KMW) to impact esthetic and peri‐implant health outcomes in the long term (Carra et al. 2023; Roccuzzo et al. 2016; Romandini, Lima, et al. 2021; Romandini, Pedrinaci, et al. 2021; Stefanini et al. 2023). Indeed, a higher STT provides greater stability of the mucosal margin (Tavelli et al. 2021), marginal bone levels compared to thinner tissues (Thoma et al. 2018), and decreases the risk of implant or prosthetic component transparency (Lops et al. 2017; Sala et al. 2017).

Different modalities have been proposed for improving STT, including bilaminar techniques using autogenous connective tissue grafts (CTG) or xenogeneic substitutes. CTG is considered the gold standard, despite being associated with patient discomfort and morbidity due to the need for a second surgical site (González‐Febles et al. 2023; Tavelli et al. 2021; Thoma et al. 2018). For these reasons, heterologous materials were introduced in periodontal and peri‐implant plastic surgery in the last decades, including a volume‐stable collagen matrix (VCMX) (Hutton et al. 2018; Lorenzo et al. 2012; Barootchi et al. 2020; Gargallo‐Albiol et al. 2019; Liu et al. 2023).

Previous studies have evaluated STA around implants, comparing VCMX and CTG mostly in esthetic areas and using different surgical approaches, stages of implant placement, assessment methods, and time points for evaluation, reporting heterogeneous results (Cairo et al. 2017; Hämmerle et al. 2023; Schmitt et al. 2021; Thoma et al. 2016; Valles et al. 2022; Zeltner et al. 2017). To the best of the authors knowledge, there is a lack of data comparing these two materials for STA simultaneous to implant placement in the presence of SBBD. Notably, one recent pilot study compared CTG to GBR around implants with SBBD, suggesting that augmenting soft tissue volume led to more stable profilometric results and comparable clinical outcomes with respect to bone augmentation (Zuercher et al. 2023). Due to the similar performance of CTG to VCMX in standard anatomical conditions, there is a rationale to test whether these concepts also apply to implants with SBBD, where the dynamics of soft/hard tissue remodeling are more penalizing (Schneider et al. 2011).

Therefore, the main purpose of the present study was to compare soft tissue volumetric and linear changes at sites augmented using VCMX or CTG at implants with SBBD (≤ 3 mm) at the time of insertion after 3 months and to evaluate the soft tissue changes at 1 year after prosthetic loading.

## 2 Materials and Methods

### 2.1 Study Design

This study was designed as a randomized clinical trial conducted with two parallel groups and a 1:1 allocation ratio. Upon approval by the local ethical committee (prot. No. 0071052), patients were recruited and screened for inclusion. The protocol of the study was registered on ClinicalTrials.gov prior to the recruitment of the first patient. The study complies with the ethical standards of the Declaration of Helsinki in 1975, revised in 2013, and it was reported according to the guidelines of the CONSORT statement (Schulz 2010).

### 2.2 Patient Selection

Patients receiving at least one implant for tooth replacement at posterior sites were consecutively selected at the section of Periodontology of C.I.R. Dental School, University of Turin, Italy from September 2022 to June 2023. The participants had to match the following criteria.

Primary inclusion criteria:

– At least 18 years old.
– Signed informed consent.
– Presence of an intercalated or distal edentulous space at both maxillary and mandibular premolar and molar areas is characterized by a Class I defect (horizontal loss of tissue with a normal apico‐coronal ridge height) (Seibert 1983).
– Tooth extraction at least 6 months earlier.
– Periodontally stable condition: no probing pocket depth (PPD) ≥ 4 mm with bleeding on probing (BoP).
– Presence of at least 4 mm of keratinized tissue.
– Good oral hygiene: full‐mouth plaque score ≤ 20% (O'Leary et al. 1972).
– Ability to comply with the study‐related procedures such as exercising good oral hygiene and attending the follow‐up procedures.

Secondary inclusion criteria (at the time of implant placement):

– Presence of SBBD ≤ 3 mm at the implant after insertion (assessed intra‐surgically).
– Adequate primary stability to allow for one‐stage implant placement (insertion torque ≥ 30 N/cm^2^).

Excluding criteria were the following:

– Pregnancy or lactation.
– Heavy smokers (> 10 cigarettes/day).
– Conditions or diseases contraindicating surgical interventions (i.e., uncontrolled diabetes mellitus), chronic use of corticosteroids or other anti‐inflammatory or immunomodulatory drugs, and drugs that affect bone metabolism or oral mucosa.
– History of stage III‐IV periodontitis (Papapanou et al. 2018).
– Previous GBR or STA surgery in the study area.

### 2.3 Sample Size Estimation

Sample size considerations were based on the primary endpoint “increase in STT at 90 days” from a previously published randomized clinical study (Zeltner et al. 2017). Expected STT in the CTG group was 0.79 mm (SD 0.45), a minimal difference of 0.4 mm between test and control treatment procedures was considered clinically relevant. The calculated sample size was 42 patients (21 per group) with a power of 80%, and alpha of 0.05. For compensation of possible dropouts (5%), 44 individuals (22 per group) were planned for enrollment.

### 2.4 Randomization and Allocation Concealment

Patients were randomly assigned to the VCMX or CTG group. A random block randomization list with a 1:1 allocation ratio was generated on a computer by an independent researcher. Notes with the assigned randomized group were enclosed in sequentially numbered, identical, opaque, and sealed envelopes by the same independent investigator and kept by an administrative employee not involved in the study. To ensure allocation concealment, the investigators involved in the selection and inclusion of the patients in the trial were blinded to the patient allocation. Every patient fulfilling eligibility criteria was consecutively enrolled and randomized to the experimental treatments. The patient envelope was opened by the nonsurgical operator only at the surgical appointment after implant placement and after the assessment of a SBBD ≤ 3 mm.

### 2.5 Surgical Phase

**FIGURE 1:**
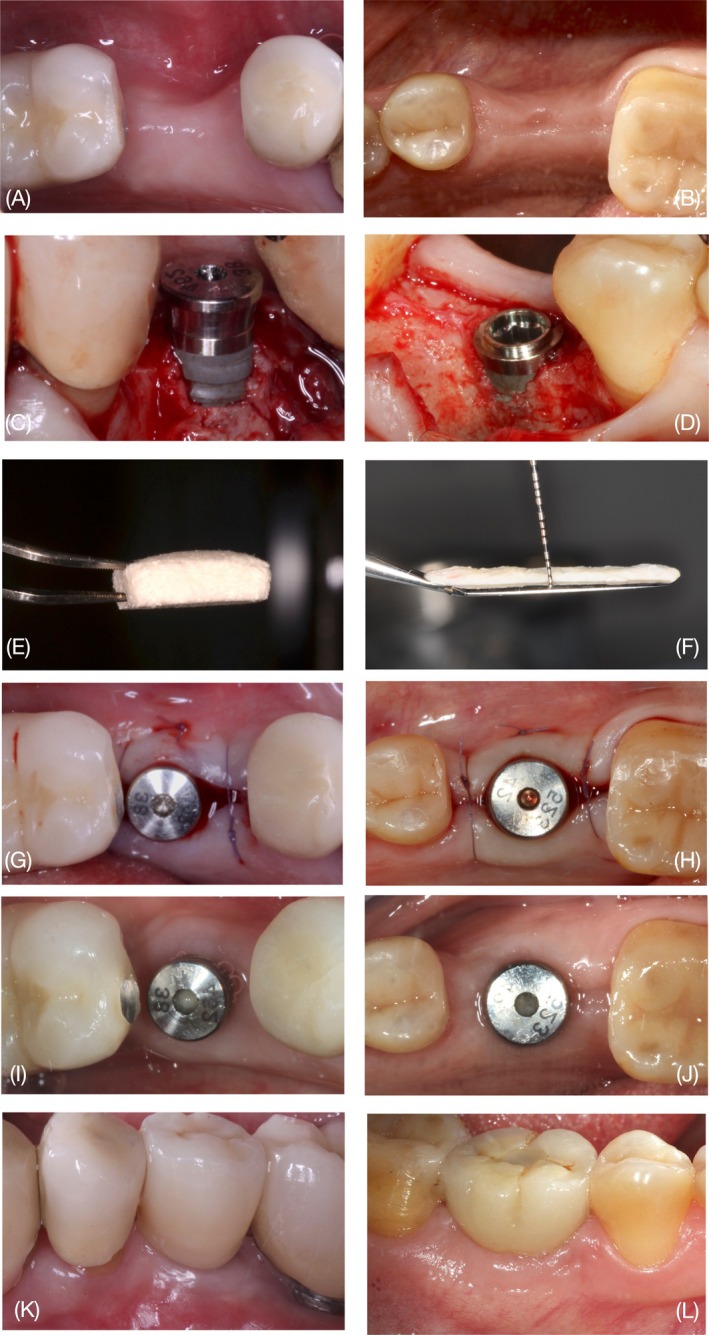
Clinical procedures represented (left: VCMX, volume‐stable collagen matrix; right: CTG, de‐epithelized connective tissue graft). (A, B) Occlusal view displaying the volume deficiency. (C, D) Intrasurgical view of small buccal bone dehiscences at the implant positioning. (E, F) VCMX and CTG harvested from the palate after trimming. (G, H) Flap stabilization with sutures and wound closure. (I, J) Occlusal view at 3 months after surgery (3 M). Clinical pictures at 1 year after prosthetic loading (K, L).

Figure 1 and Figures S1–, S4 illustrate the clinical protocol for ten representative cases. All surgeries were performed by the same operator (F.F.) with more than 20 years of experience. Immediately prior to the surgery, all patients had a mouth rinse with 0.12% chlorhexidine for 1 min. After performing local anesthesia, a horizontal crestal incision was made at the level of the edentulous ridge, always keeping at least 2 mm of keratinized tissue on the buccal side, and then extending intra‐sulcular to both the adjacent teeth. A full‐thickness flap was elevated on top of the ridge and on the lingual side. Subsequently, a partial‐thickness flap was performed at the buccal aspect extending apically beyond the muco‐gingival line, resulting in a pouch for the graft. The implant site was then prepared in order to insert a bone‐level internal connection tapered implant with a diameter of 4/4.25 mm (Certain, Biomet 3i, ZimVie, USA or Shelta, Sweden&Martina, Italy), which was immediately connected with a straight healing abutment of the same diameter. If a SBBD (< 3 mm apico‐coronally from the implant collar) was assessed, the envelope was opened, and patients were allocated to the test or control group. In the control group, a CTG was harvested from the palate and afterward de‐epithelized; in the test group a cross‐linked VCMX (25 × 25 × 6 mm; Geistlich Fibro‐Gide, Geistlich Pharma AG, Wolhusen, Switzerland) was opened. CTG and VCMX were trimmed to the desired size for the recipient bed, measured, and then stabilized in the desired position by the application of one horizontal mattress suture through the buccal flap. In both groups, a periosteal releasing incision was made to allow the grafted material to be completely covered by the flap without tension and fixed with single interrupted sutures in order to achieve closure of the augmented site. The implants were left for non‐submerged healing.

### 2.6 Postoperative Care and Follow‐Up

Patients were instructed to discontinue the home oral hygiene procedures in the surgical area after the surgical session and to rinse for 1 min with 0.2% chlorhexidine twice daily for 1 weeks. Antibiotics (amoxicillin plus clavulanic acid 1 g, twice a day for 5 days) and anti‐inflammatory drugs (ibuprofen, 600 mg) in case of need were prescribed for the postoperative phase until 1 week (Romandini et al. 2019). Fourteen days after the surgical therapy, patients returned the questionnaires, sutures were removed, and self‐performed mechanical plaque control procedures of the surgical area were reinitiated. Professional supra‐gingival teeth cleaning (using rubber cups and polishing paste) was performed, and oral hygiene instructions were reinforced at 1, 3, and 6 months after surgery (maintenance recall). Existing provisionals were adapted as necessary to avoid interference with the healing process. The final restoration was fabricated and seated on the implant according to standard clinical procedures 3 months after soft tissue grafting. The final examination took place 1 year after the delivery of the final restoration.

#### 2.6.1 Soft Tissue Changes

**FIGURE 2:**
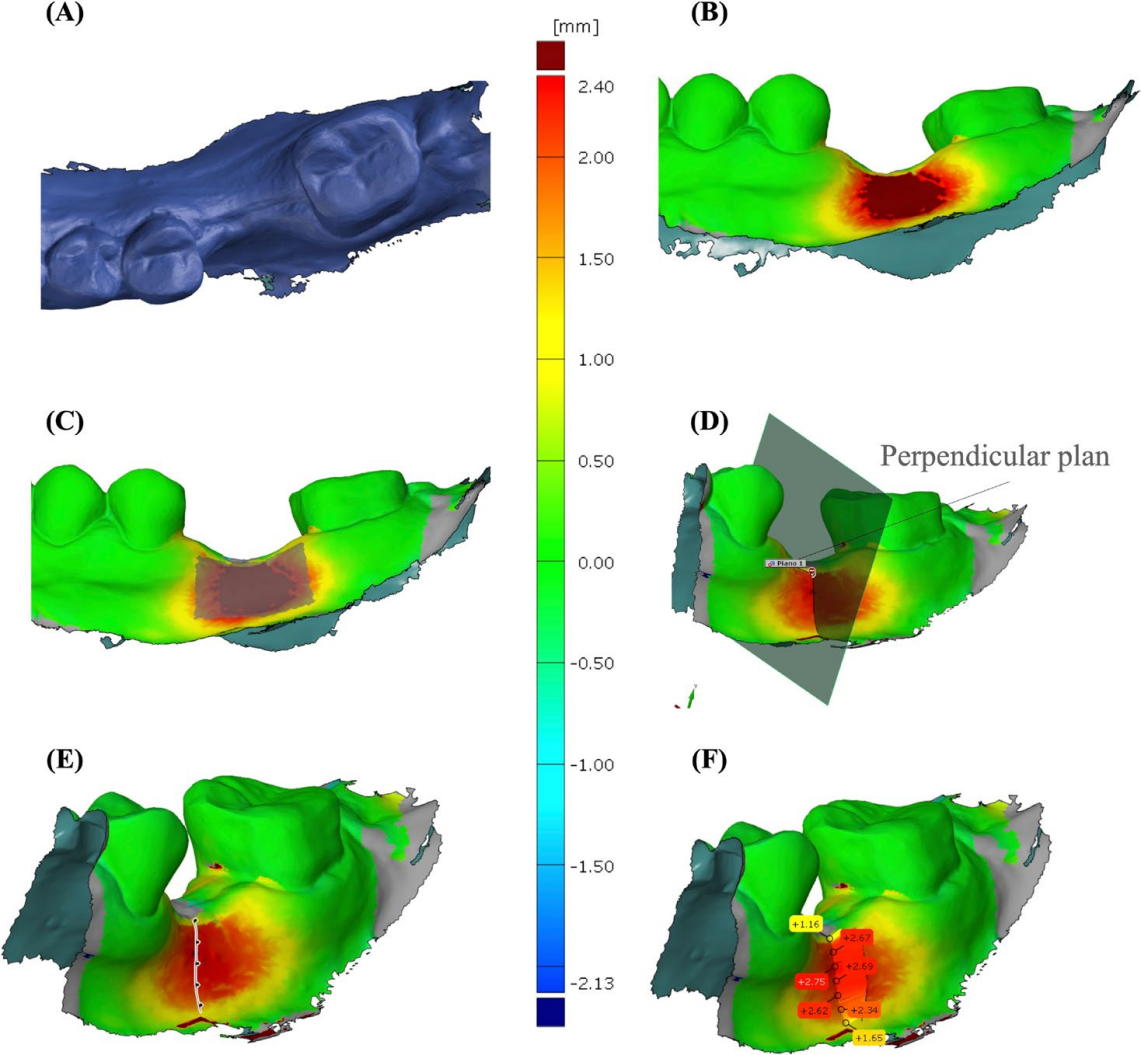
Example of volumetric and linear measurement. (A) Baseline intraoral 3D scan. (B) Superimposition of baseline and 3‐month scans. (C) The buccal region of interest (ROI) represented a trapezoidal shape located at the buccal aspect of the grafted area, starting at the crestal soft tissue margin and extending apically to the muco‐gingival border and laterally to interproximal areas. Due to the individually variable anatomical situations, the measured area varied between patients but was kept constant in each patient and site over time. (D) Perpendicular plane passing through the center of the crest. (E) Refence points at 1, 3, and 5 mm. (F) Linear measurement at the buccal profile resulting from the superimposition of the two STL files.

Prior to the surgical phase, a 3D intraoral impression encompassing the buccal and lingual/palatal aspect of the crest and at least two neighboring teeth on both sides (or a corresponding volume in case of distal edentulism) was collected. Follow‐ups were made 1 month after surgery (1 M), 3 months after surgery (3 M), and 1 year after prosthetic loading of the implant (1 Y). At 3 M, one scan was taken with the healing abutment and one with the prosthesis in place, the latter serving as a new baseline (BL‐2) to be used for the follow‐up visit at 1Y. The 3D impression obtained at baseline, 1, 3 M, and 1 Y was exported as stereolithographic (STL) files to be superimposed and matched in one common coordinate system. In order to obtain reliable measurements of the volumetric changes, a superimposition and automatic alignment of the STL files using the best‐fit algorithm was carried out with online software (GOM inspect, Braunschweig, Germany). The algorithm used the adjacent teeth surfaces to obtain a precise matching of the two considered scans. The baseline scan (baseline) was initially analyzed and superimposed on the 1 M and on the 3 M scan. Also, a superimposition was made between the 1 M compared to the 3 M scan without prosthesis, as well as between the BL‐2 and 1 Y. The volumetric changes of the vestibular soft tissues (primary outcome) were analyzed by manually selecting a region of interest (ROI) on the superimposition of the two scans selected. The ROI for evaluating tissue contour changes in a single‐implant site was selected as recommended by Tavelli et al. (2021). The ROI selected area was different among patients due to anatomical characteristics but remained constant at the patient level over the 3 months of the study, as well as over BL‐2 and 1 Y. Through these steps, it was possible to measure the volumetric change (mean Vol in mm^3^) and the area of ROI (mm^2^). Since the area varies for each site, the mean distance (∆*d* [mm] = ∆ vol [mm^3^]/area [mm^2^]) was primarily used for volumetric comparisons (Hämmerle et al. 2023). By doing so, different sites could be directly compared in terms of volume changes irrespective of their size and the size of the measured area. In the same superimposition described above, the linear variations of the vestibular contour of the soft tissues were calculated by measuring the horizontal linear distance between the two sections at 1, 3, and 5 mm from the soft‐tissue margin. Figure 2 summarizes the digital workflow of the study protocol.

#### 2.6.2 Clinical and Radiographic Measurements

For the assessment of the periodontal and peri‐implant status, the following parameters were recorded at six sites on the study implants at BL‐2 and 1‐Y, as well as on teeth adjacent to the augmented sites at BL, 1, 3 M, and 1 Y: Plaque Index (PI), keratinized tissue width (KTW), PPD, BoP, and recession. During the surgery, the following parameters were recorded: thickness of the flap with a caliper, width of the ridge, dehiscence depth, and the chair time employed. KTW was also recorded on the mid‐buccal side of the flap. The surgical procedure was also evaluated by the surgeon at the end of the session using 2 visual analogue scales (VAS) of 100 mm corresponding to the perceived difficulty and bleeding during the surgery (0 = none to 100 = worst imaginable). Surgery duration has also been recorded. Intraoral radiographs were taken at implant placement, at BL‐2, and at the 1Y follow‐up. Linear measurements of the implant marginal bone level (MBL) were measured by using the software Image J (National Institute of Health). Taking the implant shoulder as reference, negative values indicated the bone was coronal to the shoulder, while positive values were recorded when the bone was apical to the reference point. Changes over BL, BL‐2, and 1 Y were then calculated, each image being internally calibrated with the implant diameter. The average between mesial and distal measurements was obtained for each implant (Galindo‐Moreno et al. 2021).

#### 2.6.3 PROMs

Patient‐reported outcome measures (PROMs) were assessed through a questionnaire based on 100‐mm VAS scales given to the patient at the end of the surgery and returned after 2 weeks. The first point mark indicated “best sensation” and the last mark “worst imaginable sensation”. Parameters included pain, swelling and bleeding perceived at Days 0–7 and 14. Number of anti‐inflammatory drugs taken per day was also recorded.

### 2.7 Statistical Analysis

Means, standard deviations (SD), and medians (Minimum and Maximum) were obtained for a descriptive presentation of demographic parameters and outcome measures. Normal distribution of data was evaluated via the Shapiro–Wilk test. The volumetric and linear changes data were obtained as variations between baseline and 1, 1 and 3 M, BL and 3 M, as well as BL‐2 and 1 Y. *t*‐tests and Mann–Whitney *U*‐tests were used for comparing the volumetric changes and the clinical as well as radiographic outcomes between the two groups, for parametric and nonparametric variables, respectively. Categorical data were analyzed using the Chi‐square test. A *p*‐value of 5% was set for statistical significance.

## 3 Results

**FIGURE 3:**
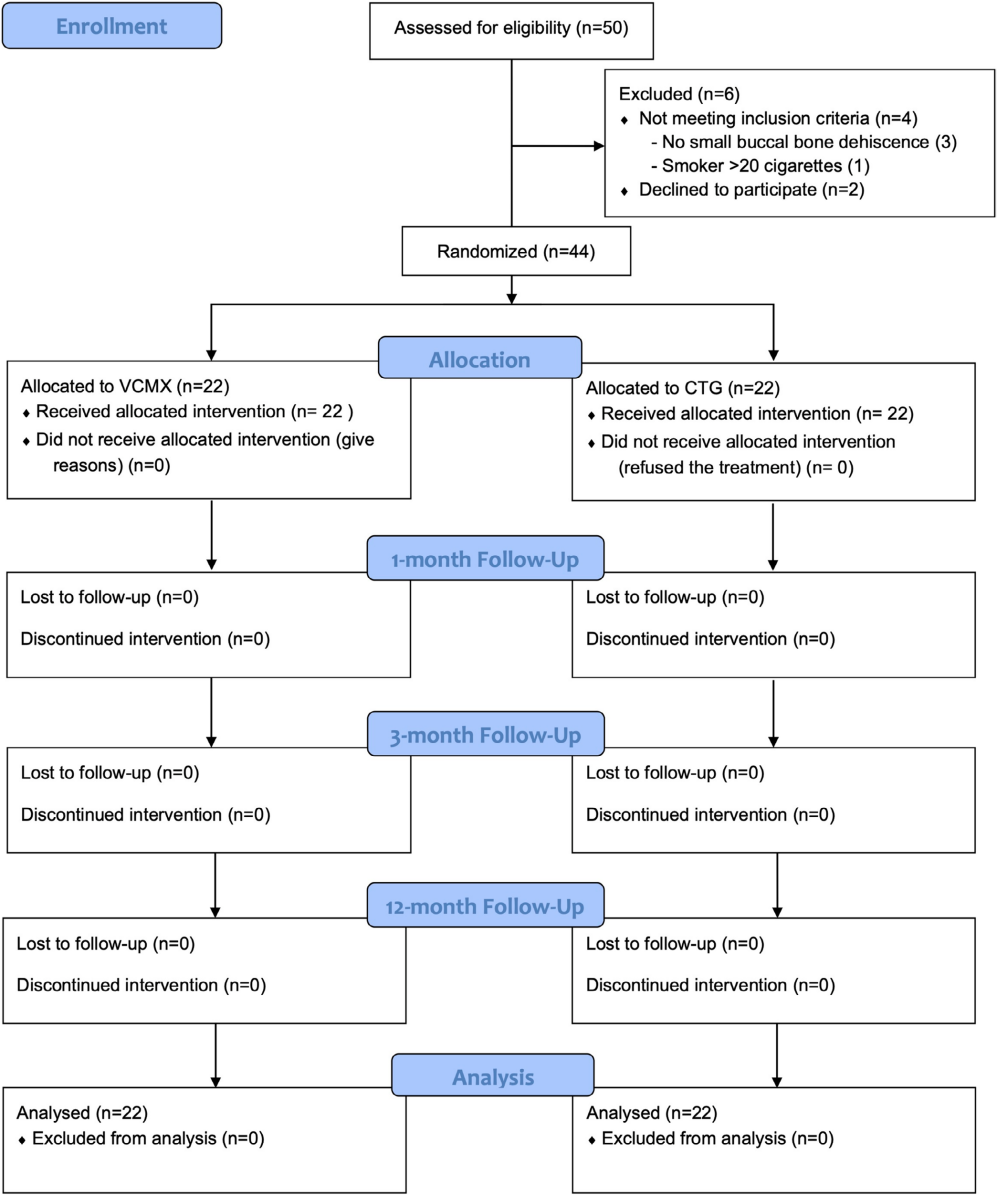
CONSORT flowchart. CTG, de‐epithelized connective tissue graft; VCMX, volume‐stable collagen matrix.

A total of 44 patients fulfilled all inclusion criteria and therefore entered the RCT. All patients underwent surgery and were analyzed at 3 months as well as 1 year after the prosthetic load, 22 in the VCMX group (test) and 22 in the CTG group (control). The CONSORT flow diagram is shown in Figure 3. No drop‐outs were experienced, and no adverse events were recorded in the postoperative phase and over the study period. An overview of baseline patient characteristics is provided in Table 1. The groups were balanced for gender, mean age, and clinical parameters at baseline.

**TABLE 1:** Patient demographics and periodontal parameters.

	VCMX (*n* = 22)	CTG (*n* = 22)
Gender		
Female, *N* (%)	12 (54.0)	11 (50.0)
Male, *N* (%)	10 (46.0)	11 (50.0)
Age (years)		
Mean ± SD	58.1 ± 11.6	61.4 ± 10.7
Jaw		
Maxillae, *N* (%)	6 (27.0)	7 (31.8)
Mandible, *N* (%)	16 (73.0)	15 (68.2)
FMPS (%)		
Mean ± SD	12.7 ± 4.8	11.9 ± 4.6
Median (Min–Max)	11.5 (5.0–20.0)	12.0 (6.0–20.0)
FMBS (%)		
Mean ± SD	12.3 ± 5.9	10.8 ± 3.7
Median (Min–Max)	12.0 (2.0–21.0)	11.0 (5.0–18.0)
Sites		
Premolars, *N* (%)	11 (50.0)	12 (54.5)
Molars, *N* (%)	11 (50.0)	10 (45.5)

Abbreviations: CTG, subepithelial connective tissue graft; FMBS, full‐mouth bleeding score; FMPS, full‐mouth plaque score; SD, standard deviation; VCMX, volume‐stable collagen matrix.

### 3.1 Clinical Outcomes

An overview of the site characteristics is presented in Table 2. Fifty‐nine percent of the implants were positioned in intercalated lacunae, while the 41% were positioned in distal lacunae. Implant sites included 13 maxilla and 31 mandibular sites. Regarding the intra‐surgical parameters, the average thickness of the flap was 1.3 mm (SD 0.3) for the control group, while it was 1.5 mm (SD 0.7) for the test group (*p* = 0.697); the average ridge width was 5.2 mm (SD 1.1) in the control group and 5.3 mm (SD 1.3) in the test group (*p* = 0.726). No significant differences in the length of the graft between the two groups were found (*p* = 0.370), but a significant difference in both thickness and height (*p* < 0.001).

**TABLE 2:** Intra‐surgical measurements and surgeon perception of the clinical procedure (VAS).

	VCMX (*n* = 22)	CTG (*n* = 22)	*p*
Flap thickness, mm			
Mean ± SD	1.5 ± 0.7	1.3 ± 0.3	0.697^b^
Median (Min–Max)	1.4 (0.3–3.0)	1.2 (0.7–2.1)	
KT baseline, mm			
Mean ± SD	2.82 ± 1.25	2.55 ± 1.27	0.484^b^
Median (Min–Max)	3.0 (1.0–5.0)	2.0 (0.0–5.0)	
Ridge width, mm			
Mean ± SD	5.3 ± 1.3	5.2 ± 1.1	0.726^b^
Median (Min–Max)	5.0 (4.0–8.0)	5.0 (4.0–8.0)	
Buccal bone dehiscence, mm			
Mean ± SD	2.4 ± 0.7	2.3 ± 0.9	0.381^b^
Median (Min–Max)	2.0 (1.0–3.0)	2.5 (1.0–3.0)	
Graft width, mm			
Mean ± SD	14.5 ± 4.1	13.3 ± 4.6	0.370^b^
Median (Min–Max)	13.5 (8.0–20.0)	12.0 (7.0–22.0)	
Graft height, mm			
Mean ± SD	6.7 ± 1.3	5.2 ± 0.8	**< 0.001** ^b^
Median (Min–Max)	6.0 (4.0–10.0)	5.0 (4.0–7.0)	
Graft thickness, mm			
Mean ± SD	5.9 ± 0.3	1.7 ± 0.7	**< 0.001** ^b^
Median (Min–Max)	6.0 (5.0–6.0)	1.5 (1.0–4.0)	
Surgery duration, min			
Mean ± SD	42.5 ± 14.2	47.7 ± 8.9	0.154^a^
Median (Min–Max)	37.5 (25.0–75.0)	46.0 (35.0–75.0)	
Difficulty of the surgery (VAS scale)			
Mean ± SD	15.5 ± 6.7	28.9 ± 14.3	**< 0.001** ^b^
Median (Min–Max)	15.0 (5.0–35.0)	29.0 (1.0–55.0)	
Bleeding during surgery (VAS scale)			
Mean ± SD	13.1 ± 6.1	25.7 ± 13.1	**< 0.001** ^b^
Median (Min–Max)	12.0 (6.0–32.0)	24.5 (9.0–60.0)	

*Note:* Bold indicates the statistically significant difference.

Abbreviations: SD, standard deviation; VAS, visual analog scale.

^a^ Student *t*‐test.

^b^ Mann–Whitney *U*‐test.

The average duration of surgery was 42.5 min (SD 14.2) for the test group, while for the control group, it was 47.7 min (SD 8.9) (*p* = 0.154). The perception of the difficulty of the surgery, measured through a VAS scale of 100, was 28.9 (SD 14.3) for the CTG group and 15.5 (SD 6.7) for the VCMX group. The perception of the bleeding was 25.7 (SD 13.1) for the control group and 13.1 (SD 6.1) for the test group. A significant difference between the two groups was detected for both parameters (*p* < 0.001).

At BL‐2, the clinical data recorded at the mid‐buccal site of the new prosthesis reflected a mean PPD of 2.7 mm (SD 0.8) in the CTG group and 2.8 mm (SD 0.7) in the VCMX group (*p* = 0.760). The mean KTW was significantly higher in the CTG group than in the VCMX group (3.9 mm [SD 1.0] vs. 3.2 mm [SD 1.1], respectively; *p* = 0.033). At 1Y, the mean PPD was 2.5 mm (SD 0.6) for the CTG and 2.3 mm (SD 0.7) for the VCMX (*p* = 0.338). Again, KTW was 3.7 mm (SD 1.5) in the CTG and 2.8 mm (SD 1.0) in the VCMX (*p* = 0.042), whereas mucosal recession from the BL‐2 reference point was 0.14 mm (SD 0.47) in the CTG and 0.05 mm (SD 0.21) in the VCMX (*p* = 0.537). At both time points, PI and BoP at implant sites varied between 0% and 15%. All other parameters on adjacent teeth did not display any significant differences within or between groups.

### 3.2 Volumetric and Linear Changes From BL to 1 M

The descriptive measures for the volumetric and linear changes throughout the experimental period are presented in Table 3. Between baseline and 1 M, all patients displayed a volume gain. In terms of mean distance, no difference was observed between the two groups, with 1.77 mm (SD 0.54) for VCMX and 1.54 mm (SD 0.50) for CTG (*p* = 0.206). For the change in mean volume, the augmentation amounted to 81.87 mm^3^ (SD 27.47) for the test group and 79.17 mm^3^ (SD 28.84) for the control group. The changes between BL and 1 M did not reveal a statistically significant difference between the test and control groups in terms of linear measurements at 1 mm (*p* = 0.883), 3 mm (*p* = 0.441), and 5 mm (*p* = 0.280).

**TABLE 3:** Linear and volumetric changes.

	VCMX (*n* = 22)		CTG (*n* = 22)		*p* between group
	Mean ± SD	Median (Min–Max)	Mean ± SD	Median (Min–Max)	
BL to 1 M					
Mean distance (mm)	1.77 ± 0.54	1.76 (0.63–2.88)	1.54 ± 0.50	1.50 (0.48–2.96)	0.150
Mean volume (mm^3^)	81.87 ± 27.47	84.08 (28.32–134.50)	79.17 ± 28.84	79.23 (26.82–135.16)	0.752
Linear 1 mm (mm)	1.80 ± 0.74	1.75 (0.41–2.90)	1.77 ± 0.61	1.90 (0.5–2.70)	0.883
Linear 3 mm (mm)	2.18 ± 0.66	2.09 (0.96–3.39)	2.03 ± 0.61	1.92 (0.80–3.20)	0.441
Linear 5 mm (mm)	2.15 ± 0.62	2.02 (0.85–3.38)	1.91 ± 0.78	1.91 (0.38–3.61)	0.280
1 to 3 M					
Mean distance (mm)	−0.61 ± 0.53	−0.46 (−1.64 to 0.08)	−0.29 ± 0.30	−0.18 (−0.87 to 0.17)	**0.022**
Mean volume (mm^3^)	30.64 ± 16.68	28.56 (5.50–65.12)	25.22 ± 32.52	13.23 (2.88–133.94)	0.491
Linear 1 mm (mm)	−0.35 ± 0.40	−0.27 (−1.47 to 0.32)	−0.04 ± 0.41	−0.09 (−0.76 to 1.32)	**0.015**
Linear 3 mm (mm)	−0.68 ± 0.41	−0.64 (−1.56 to 0.10)	−0.19 ± 0.43	−0.19 (−1.23 to 2.47)	**0.004**
Linear 5 mm (mm)	−0.93 ± 0.54	−0.89 (−2.1 to 0.24)	−0.17 ± 0.90	−0.35 (−1.04 to 2.49)	**0.002**
BL to 3 M					
Mean distance (mm)	1.07 ± 0.22	1.08 (0.49–1.43)	1.22 ± 0.44	1.18 (0.32–2.21)	0.156
Mean volume (mm^3^)	46.56 ± 13.21	46.72 (26.72–86.55)	58.84 ± 29.33	50.22 (15.07–136.26)	0.084
Linear 1 mm (mm)	1.42 ± 0.46	1.44 (0.32–2.27)	1.52 ± 0.62	1.52 (0.30–2.67)	0.569
Linear 3 mm (mm)	1.41 ± 0.36	1.41 (0.77–2.31)	1.69 ± 0.50	1.68 (0.8–2.69)	**0.041**
Linear 5 mm (mm)	1.24 ± 0.39	1.3 (0.53–1.80)	1.48 ± 0.61	1.35 (0.38–2.57)	0.137
BL‐2 to 1 Y					
Mean distance (mm)	−0.05 ± 0.36	−0.05 (−0.91 to 1.09)	0.21 ± 0.32	0.15 (−0.19 to 1.30)	**0.014**
Mean volume (mm^3^)	47.03 ± 2.73	46.58 (41.23–51.23)	46.37 ± 3.06	46.35 (41.21–50.65)	0.451
Linear 1 mm (mm)	0.22 ± 0.37	0.23 (−0.84 to 0.82)	0.38 ± 0.29	0.38 (−0.09 to 1.28)	0.116
Linear 3 mm (mm)	−0.03 ± 0.31	0.09 (−0.92 to 0.48)	0.24 ± 0.30	0.18 (−0.34 to 1.09)	**0.005**
Linear 5 mm (mm)	−0.20 ± 0.28	−0.19 (−0.80 to 0.49)	0.08 ± 0.34	0.10 (−0.43 to 0.80)	**0.006**

*Note: p* values were calculated using Student *t*‐test.

Abbreviations: 1 Y, follow‐up at 1 year after implant loading; BL, baseline; BL‐2, placement of final restoration at 3 months; M, month; SCTG, subepithelial connective tissue graft; SD, standard deviation; VCMX, volume‐stable collagen matrix.

### 3.3 Volumetric and Linear Changes From 1 to 3 M

Between 1 and 3 M, there was an overall profilometric volume loss in all cases, except for one patient in the CTG group where it remained stable. The mean distance between 1 and 3 M decreased more in the VCMX group than in the control group (0.61 mm [SD 0.53] vs. 0.29 mm [SD 0.30]; *p* = 0.022), while the mean volume did not show statistical significance (*p* = 0.491). Consistently, the mean linear change revealed a more significant reduction for the VCMX at 1, 3, and 5 mm (*p* = 0.015, *p* = 0.004 and 0.002, respectively).

### 3.4 Volumetric and Linear Changes From BL to 3 M

Despite the slight shrinkage observed between 1 and 3 M, there was a net profilometric volume gain in all cases at 3 M. The mean distance (primary endpoint) between BL and 3 M was 1.07 mm (SD 0.22) for the VCMX group and 1.22 mm (SD 0.44) for the CTG group (*p* = 0.156). Also, the mean volume revealed no statistically significant difference at 3 months between the two groups (46.56 mm^3^ [SD 13.21] for the test group and 58.84 mm^3^ [SD 29.33] for the control group, *p* = 0.084). Linear changes for VCMX application at 1, 3, and 5 mm were 1.42 mm (SD 0.46), 1.41 mm (SD 0.36) and 1.24 mm (SD 0.39), respectively. When using the CTG, the corresponding figure at 1, 3, and 5 mm was 1.52 mm (SD 0.62), 1.69 mm (SD 0.50), and 1.48 mm (SD 0.61), respectively. For both groups, the soft tissue increase was statistically significant (*p* < 0.01). The difference between groups resulted in a statistically significant difference only at 3 mm from the gingival margin (*p* = 0.041).

### 3.5 Volumetric and Linear Changes From BL‐2 to 1Y

At the last follow‐up between prosthesis delivery and 1Y, the VCMX group experienced a slight decrease in the mean distance value, whereas CTG provided more stability (−0.05 mm [SD 0.36] vs. 0.21 mm [SD 0.32], respectively; *p* = 0.014). Conversely, the mean volume revealed no statistically significant difference between the two groups (47.03 mm^3^ [SD 2.73] for the test group and 46.37 mm^3^ [SD 3.06] for the control group, *p* = 0.084). As for linear measures, statistically significant improvements were observed at 3 mm (*p* = 0.005) and 5 mm (*p* = 0.006) favoring the CTG group.

### 3.6 Radiographic Outcomes

Average MBL change from loading to the 1‐year follow‐up was −0.34 mm (SD 0.70) versus 0.09 mm (SD 0.65) for the VCMX and CTG groups, respectively; the difference was statistically significant (*p* < 0.05). Conversely, all the other radiographic measures between groups were not differing significantly (Table S1).

### 3.7 PROMs

**FIGURE 4:**
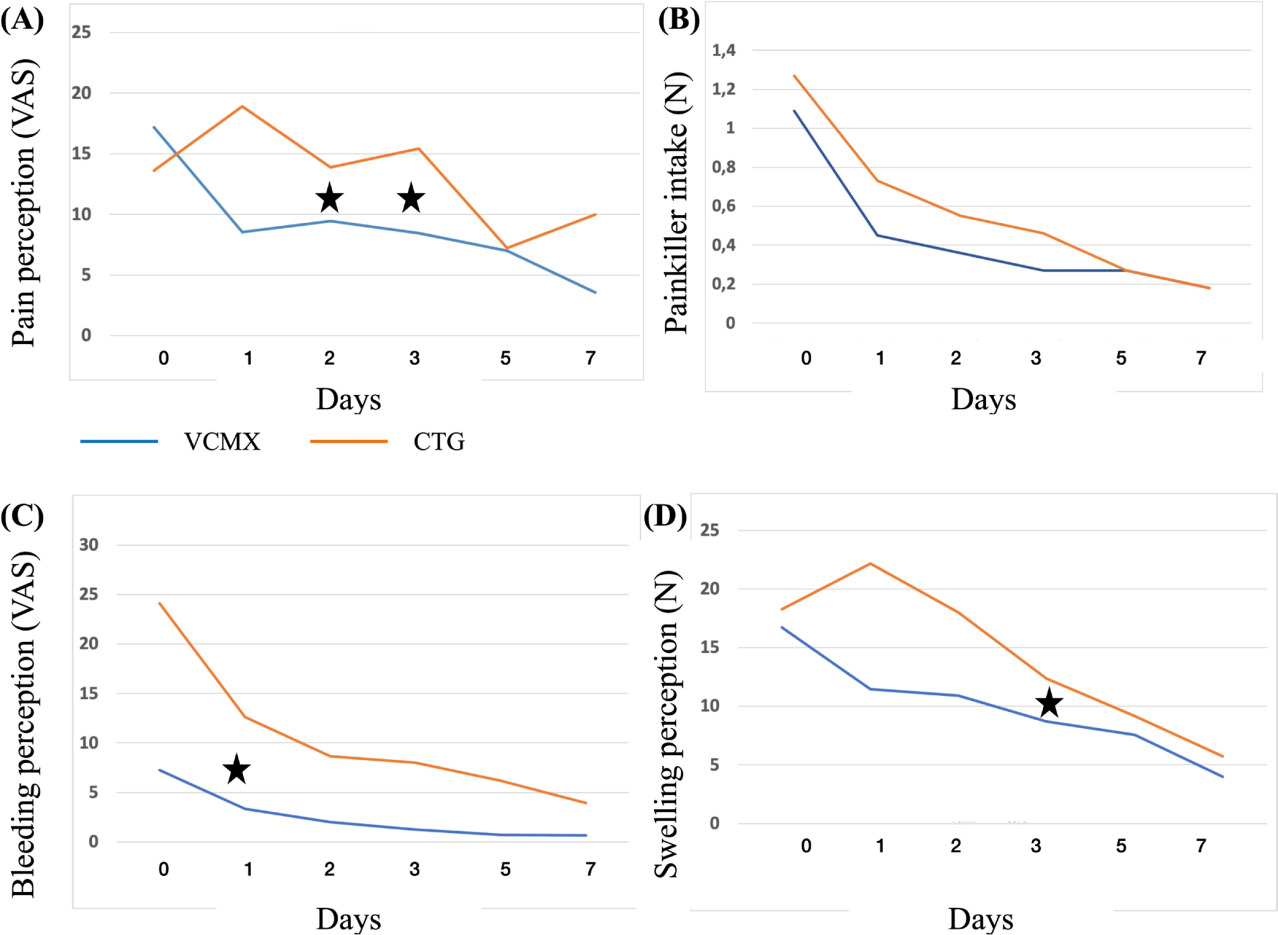
Patient‐reported outcome measures (PROMs) after the surgery represented as line plots (VAS 0–100). Orange line: Subepithelial connective tissue graft (SCTG); Blue line: Volume‐stable collagen matrix (VCMX). The report shows pain (A), drug assumption (B), bleeding (C), and swelling (D) in both treatment groups (VCXM blue; CTG orange) on a 7 days period of healing. Patients in the VCMX group reported significantly less bleeding at Day 1, less pain at Days 2 and 3 and swelling at Days 3 and 5 compared to the CTG group. Black stars show statistically significant difference between groups (*p* < 0.05).

Figure 4 shows a visual representation of PROMs during the first week after the intervention, recorded on a visual analog scale from 0 to 100. At Day 0, pain had a mean of 13.7 (SD 18.7) for the CTG group and a mean of 7.8 (SD 9.7) for the VCMX group. Swelling had a mean of 12.5 (SD 17.5) for CTG and a mean of 6.7 (SD 9.9) for VCMX, while bleeding had a mean of 1.1 (SD 1.2) for the control group and a mean of 0.7 (SD 0.9) for the test group. The statistical analysis showed a statistically significant difference in the perception of the bleeding at Day 1 (*p* = 0.042), pain at 2 days (*p* = 0.047) and 3 days (*p* = 0.046), and swelling at 3 days (p = 0.042). The mean weekly intake of anti‐inflammatory drugs was 3.1 (SD 6.3) for CTG and 1.8 (SD 4.6) for VCMX, showing no statistically significant difference (*p* = 0.518). The perception of the surgery, assessed on a visual analog scale from 0 to 100, was also not statistically significant between the two groups (*p* = 0.131). Finally, regarding esthetic perception at the treated sites, patients perceived an improvement between baseline and 3 months, with no statistically significant differences between the two groups (*p* = 0.270).

## 4 Discussion

The present RCT was performed to compare VCMX versus CTG (gold‐standard) for buccal STA simultaneously to one‐stage implant placement at posterior sites with small buccal bone dehiscences. This augmentation was evaluated both by volumetric and linear measurements performed by the superimposition of the 3D intraoral scans. Both VCMX and CTG showed uneventful healing and clinical integration, resulting in an increase in the volumetric and linear ridge at 3 M and 1 Y. These findings confirm that both CTG and VCMX are effective in STA around implants, even in the presence of SBBD, with the former option demonstrating a more stable profile at the more extended follow‐up. No differences were noted regarding clinical peri‐implant parameters, whereas radiographic MBL appeared more maintained after CTG application. Finally, lower pain perception, bleeding, and swelling were experienced with VCMX in the short term.

It is generally accepted that soft tissue grafting procedures provide improved clinical, esthetic, and radiographic outcomes for implant therapy (Stefanini et al. 2023; Thoma et al. 2018). In terms of STA evaluation, the present values regarding the VCMX versus CTG comparison are in line with previous investigations. Cairo et al. (2017) evaluated CTG against a collagen matrix applied in a second‐stage procedure (uncovering of the implants) after 6 months of healing. In this study, CTG resulted more effectively than the matrix to increase buccal peri‐implant STT measured using an injection needle. Also, Thoma et al. (2016) conducted a RCT on STA around implants that were randomly assigned to VCMX or CTG in a submerged healing technique. Comparable results at 90 days were obtained in terms of STA between test and control groups using an endodontic file with an individualized stent. At the 5‐year follow‐up, peri‐implant health was maintained in both groups with stable clinical and radiographic outcomes (Thoma et al. 2023). Similarly, Zeltner et al. (2017) published a RCT in which 20 patients underwent STA at implant sites with a CTG or a VCMX, considering only linear changes and including both buccal and crestal areas assessed on model casts. At 3 months, the mean distance was 0.79 (SD 0.45) mm for the CTG and 0.77 (SD 0.74) mm for the VCMX. More recently, STA with either VCMX or CTG was performed simultaneously to the implant placed with a submerged healing and using 3D scans superimpositions to determine the degree of volume augmentation (Hämmerle et al. 2023). In both groups, the authors observed positive volumetric changes between baseline and 3 months after surgery (0.9 mm for the VCMX and 1.1 for the CTG), which were slightly inferior to the results obtained in the present study. These comparisons corroborate a consistent efficacy of both graft materials in gaining STT at implant sites. The minor differences observed may be due to heterogeneous surgical protocols used, timings of STA procedures, and outcome assessment methods.

Notably, all these aforementioned studies evaluated implants without dehiscence or fenestration defects at the time of insertion. Regarding SBBDs, one RCT demonstrated that failure to address this problem by GBR can lead to an increase in vertical bone loss (Jung et al. 2017), as well as impact the aesthetic outcome of implant therapy and the overall satisfaction of patients (Thoma et al. 2021). Recently, the use of CTG has been proposed as a viable alternative to GBR, due to its higher dimensional stability and the capacity to yield comparable clinical results (Thoma et al. 2019). Notably, one recent pilot study compared STA with CTG against GBR for the management of implants placed with SBBD (Zuercher et al. 2023), with the former resulting in comparable clinical outcomes, but more stable profilometric outcomes. This suggests that STA can be considered in relatively critical anatomical conditions, despite the underlying mechanisms requiring further corroboration.

When studying the effect of STA, reliable methods for the volumetric comparisons at different time points are needed (Dhondt et al. 2022; Figueredo et al. 2024). Transgingival probing is a traditional method for assessing STT, and it is typically measured 1 to 3 mm apical to the soft tissue margin (Hutton et al. 2018). However, this measurement represents the mucosal thickness variation at a specific reference point (linear changes), which may not be representative of the overall volume gain/loss after surgery (Barootchi et al. 2020). On the other hand, digital technologies based on optical scanning can generate 3D digital models intra‐orally or from scanning conventional dental casts. The superimposition of digital STL files obtained by 3D intraoral scans at different time points for the assessment of volume changes represents a well‐established method that has been applied in both preclinical and clinical studies to evaluate the efficacy of STA interventions both in terms of linear and volumetric changes, as well as for monitoring tissue stability over time (Bienz et al. 2017; Naenni et al. 2021; Papi et al. 2021; Tavelli et al. 2021; Tavelli and Barootchi 2024). Although the accuracy of the intraoral digital impression is strongly affected by operator's learning curve and the size of the scanned area, the reliability of this method has been proven (Bosniac et al. 2019).

In terms of secondary outcomes, some potentially clinically relevant observations emerged from the present RCT. At both 3 M and 1 Y, KTW resulted significantly higher in the CTG group than the VCMX. Also, the radiographic comparisons between BL‐2 and 1 Y showed a slightly more maintained peri‐implant bone profile in the CTG group. These data lead to speculate some biologically mediating effects for CTG with respect to its inert counterpart VCMX, which warrant further research investigations. Lastly, few notable differences between the groups in terms of PROMs were recorded. The statistically significant advantage of using VCMX over CTG was only observed in terms of bleeding at Day 1, pain at Days 2 and 3, and swelling at 3 days. This partially contrasts with previous investigations that have proposed the superiority of the xenogenic matrix over CTG based on reduced painkiller intake, postoperative discomfort, and total chair time (Cairo et al. 2017). Despite PROMs being highly subjective, the postoperative experience of CTG seems to be also influenced by the thickness of the harvested tissue. Indeed, it has been assessed that patient perception of pain was positively correlated with the depth of the wound at the donor site, whereas the wound surface area did not have a major impact (Del Pizzo et al. 2002). Notably, the length of the surgical intervention did not differ between the two groups. These findings agree with the recent RCT by Hämmerle et al. (2023), but disagree with those reported in previous literature (Thoma et al. 2023). Apart from operator experience in CTG harvesting, this might be explained by the time needed for trimming and stabilizing the VCMX. Regarding surgeon perception, relevant differences were indeed found, since the perceived difficulty was higher in the CTG group, as well as the bleeding perceived during it (*p* < 0.001).

To our knowledge, this is the first study to analyze STA simultaneously to implant positioning with SBBD, conducting both a volumetric and a linear soft tissue analysis using 3D model casts superimposition. Importantly, the protocol was implemented at posterior areas and in subjects with no/low susceptibility to periodontitis, with potentially different results expected in patients with a periodontitis history, especially in the long term. However, the present study has some limitations. First, despite a sample size calculation being conducted based on the primary outcome leading to significantly different results, increasing the numerosity might have allowed us to stratify the outcomes according to the location of the augmented site (intercalated or distal) or to correlate volumetric outcomes with clinical and radiographic features. Secondly, the thickness of CTG was not standardized. However, clinical effort in achieving a homogeneous layer between patients was attempted, despite the inherent defect variations and the anatomy of the donor site. Finally, the monocentric nature of the investigation may limit its external generalizability.

## 5 Conclusion

Within the limits of the present analysis, STA using either CTG or VCMX resulted in a consistent augmentation of buccal soft tissues at posterior implant sites with SBBD at 3 months, which was maintained after 1 year of loading in patients not susceptible to periodontitis. The use of CTG provided slightly improved results compared to VCMX in terms of both volumetric and linear outcomes, while clinical peri‐implant data were similar. Also, CTG allowed for a better radiographic MBL stability in the first year after loading and higher KTW. Finally, PROMs revealed statistically significant differences in the perception of the bleeding at day 1, pain at 2 and 3 days, and swelling at 3 days favoring the VCMX. Long‐term clinical studies after the prosthetic load and considering clinical outcomes related to peri‐implant health are required to further validate these findings.

## Author Contributions

**Francesco Ferrarotti:** conceptualization, investigation, methodology, supervision, writing – review and editing. **Giacomo Baima:** conceptualization, investigation, writing – original draft, methodology, visualization, software, data curation, supervision. **Giulia Mohammadi:** investigation, methodology, software, visualization, writing – original draft. **Clelia Carboncini:** software, data curation, investigation, writing – original draft. **Federica Romano:** writing – review and editing, conceptualization, investigation, methodology, validation, formal analysis, supervision, data curation. **Mario Aimetti:** conceptualization, investigation, writing – review and editing, project administration, supervision, resources.

## Ethics Statement

The study protocol was in accordance with the Declaration of Helsinki, approved by the Ethics Committee of the A.O.U. Città della Salute e della Scienza of Turin, Italy (protocol number 0041679; approval date 15/4/2021).

## Conflicts of Interest

The authors declare no conflicts of interest.

## Supporting information


**Table S1.** Description and comparison of radiographical variables (mean ± SD).

**Figure S1.** Clinical procedures represented (left: VCMX, volume‐stable collagen matrix; right: CTG, de‐epithelized connective tissue graft). (A, B) Occlusal view displaying the volume deficiency. (C, D) Intrasurgical view of small buccal bone dehiscences at the implant positioning. (E, F) VCMX and CTG harvested from the palate after trimming. (G, H) Flap stabilization with sutures and wound closure. (I, J) Occlusal view at 3 months after surgery (3 M). Clinical pictures at 1 year after prosthetic loading (K, L).

**Figure S2.** Clinical procedures represented (left: VCMX, volume‐stable collagen matrix; right: CTG, de‐epithelized connective tissue graft). (A, B) Occlusal view displaying the volume deficiency. (C, D) Intrasurgical view of small buccal bone dehiscences at the implant positioning. (E, F) VCMX and CTG harvested from the palate after trimming. (G, H) Flap stabilization with sutures and wound closure. (I, J) Occlusal view at 3 months after surgery (3 M). Clinical pictures at 1 year after prosthetic loading (K, L).

**Figure S3.** Clinical procedures represented (left: VCMX, volume‐stable collagen matrix; right: CTG, de‐epithelized connective tissue graft). (A, B) Occlusal view displaying the volume deficiency. (C, D) Intrasurgical view of small buccal bone dehiscences at the implant positioning. (E, F) VCMX and CTG harvested from the palate after trimming. (G, H) Flap stabilization with sutures and wound closure. (I, J) Occlusal view at 3 months after surgery (3 M). Clinical pictures at 1 year after prosthetic loading (K, L).

**Figure S4.** Clinical procedures represented (left: VCMX, volume‐stable collagen matrix; right: CTG, de‐epithelized connective tissue graft). (A, B) Occlusal view displaying the volume deficiency. (C, D) Intrasurgical view of small buccal bone dehiscences at the implant positioning. (E, F) VCMX and CTG harvested from the palate after trimming. (G, H) Flap stabilization with sutures and wound closure. (I, J) Occlusal view at 3 months after surgery (3 M). Clinical pictures at 1 year after prosthetic loading (K, L).

## Data Availability

The data that support the findings of this study are available from the corresponding author upon reasonable request.
