# Supplementary material for: Peri‐Implant Soft Tissue Increase at Small Buccal Bone Dehiscences With Either Volume‐Stable Collagen Matrix or Connective Tissue Graft: A Randomized Controlled Trial
Source: Clin Oral Implants Res. 2025 Mar 19;36(7):846–58. doi: 10.1111/clr.14430 (PMC12230890; doi:10.1111/clr.14430)
Supplement: Supplementary file 1 — Table S1. Description and comparison of radiographical variables (mean ± SD). [file CLR-36-846-s001.docx]

**Supplementary Table 1**. Description and comparison of radiographical variables (mean ± *SD*).

|  | BL | 3M | 1Y | Δ baseline-3M | Δ baseline-1Y | Δ 3M-1Y* |
| --- | --- | --- | --- | --- | --- | --- |
| VCMX (n=22) | 0.34 ± 0.72 | 1.10 ± 0.79 | 1.44 ± 1.01 | -0.76 ± 0.61 | -1.11 ± 0.76 | -0.34 ± 0.70 |
| CTG (n=22) | 0.47 ± 0.64 | 1.43 ± 1.00 | 1.34 ± 1.23 | -0.96 ± 0.93 | -0.87 ± 1.18 | 0.09 ± 0.65 |

Abbreviations. VCMX, volume-stable collagen matrix; SCTG, subepithelial connective tissue graft; BL, implant insertion; 3M, placement of final restoration at 3 months; 1Y, follow-up at 1 year after implant loading; SD, standard deviation.

*p<0.05 between groups, Mann Whitney U-test
