# Supplementary figures and images for: Peri‐Implant Soft Tissue Increase at Small Buccal Bone Dehiscences With Either Volume‐Stable Collagen Matrix or Connective Tissue Graft: A Randomized Controlled Trial
Source: Clin Oral Implants Res. 2025 Mar 19;36(7):846–58. doi: 10.1111/clr.14430 (PMC12230890; doi:10.1111/clr.14430)

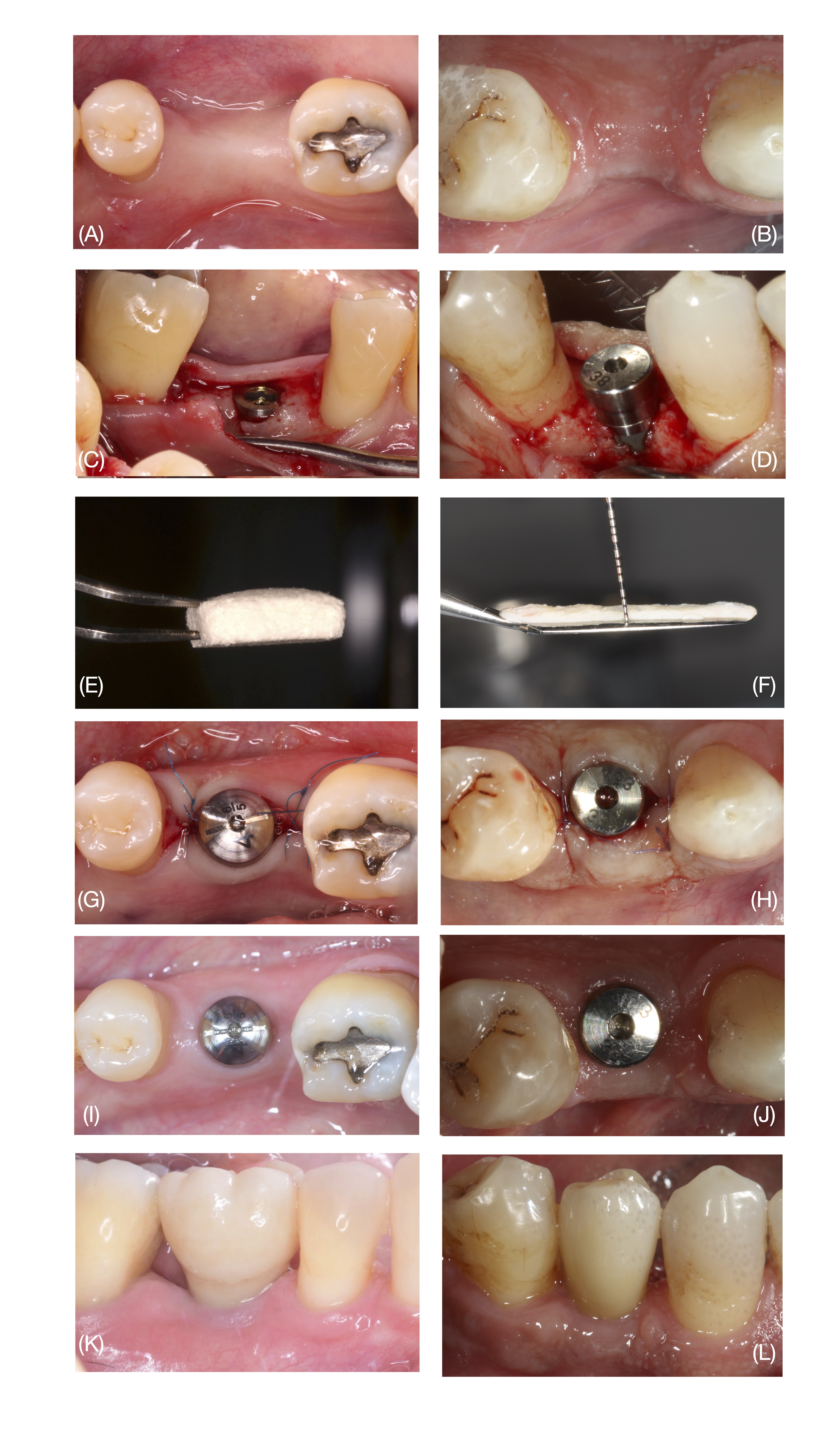

Supplement: Supplementary file 2 — Figure S1. Clinical procedures represented (left: VCMX, volume‐stable collagen matrix; right: CTG, de‐epithelized connective tissue graft). (A, B) Occlusal view displaying the volume deficiency. (C, D) Intrasurgical view of small buccal bone dehiscences at the implant positioning. (E, F) VCMX and CTG harvested from the palate after trimming. (G, H) Flap stabilization with sutures and wound closure. (I, J) Occlusal view at 3 months after surgery (3 M). Clinical pictures at 1 year after prosthetic loading (K, L). [file CLR-36-846-s003.tiff]

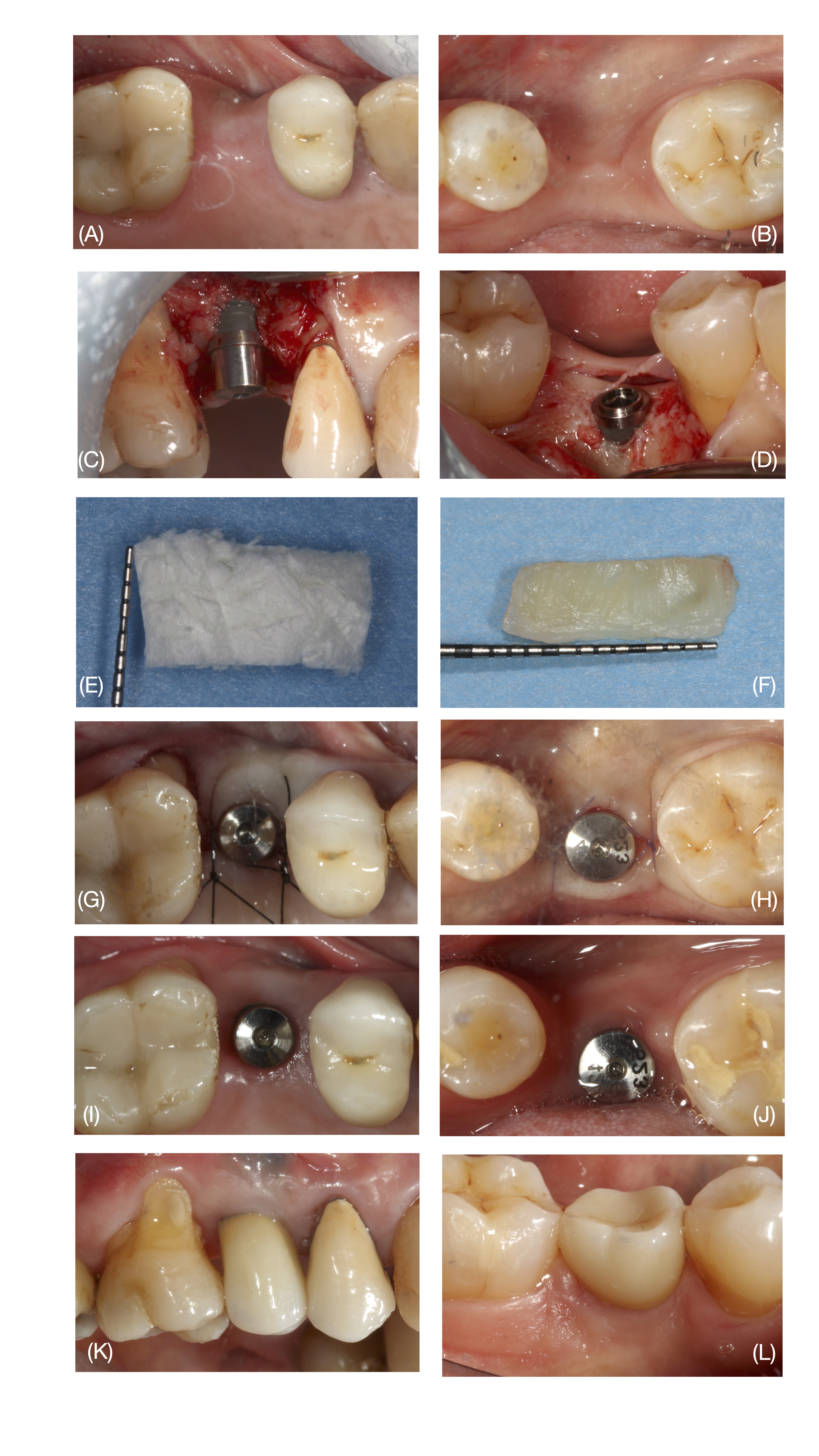

Supplement: Supplementary file 3 — Figure S2. Clinical procedures represented (left: VCMX, volume‐stable collagen matrix; right: CTG, de‐epithelized connective tissue graft). (A, B) Occlusal view displaying the volume deficiency. (C, D) Intrasurgical view of small buccal bone dehiscences at the implant positioning. (E, F) VCMX and CTG harvested from the palate after trimming. (G, H) Flap stabilization with sutures and wound closure. (I, J) Occlusal view at 3 months after surgery (3 M). Clinical pictures at 1 year after prosthetic loading (K, L). [file CLR-36-846-s004.tiff]

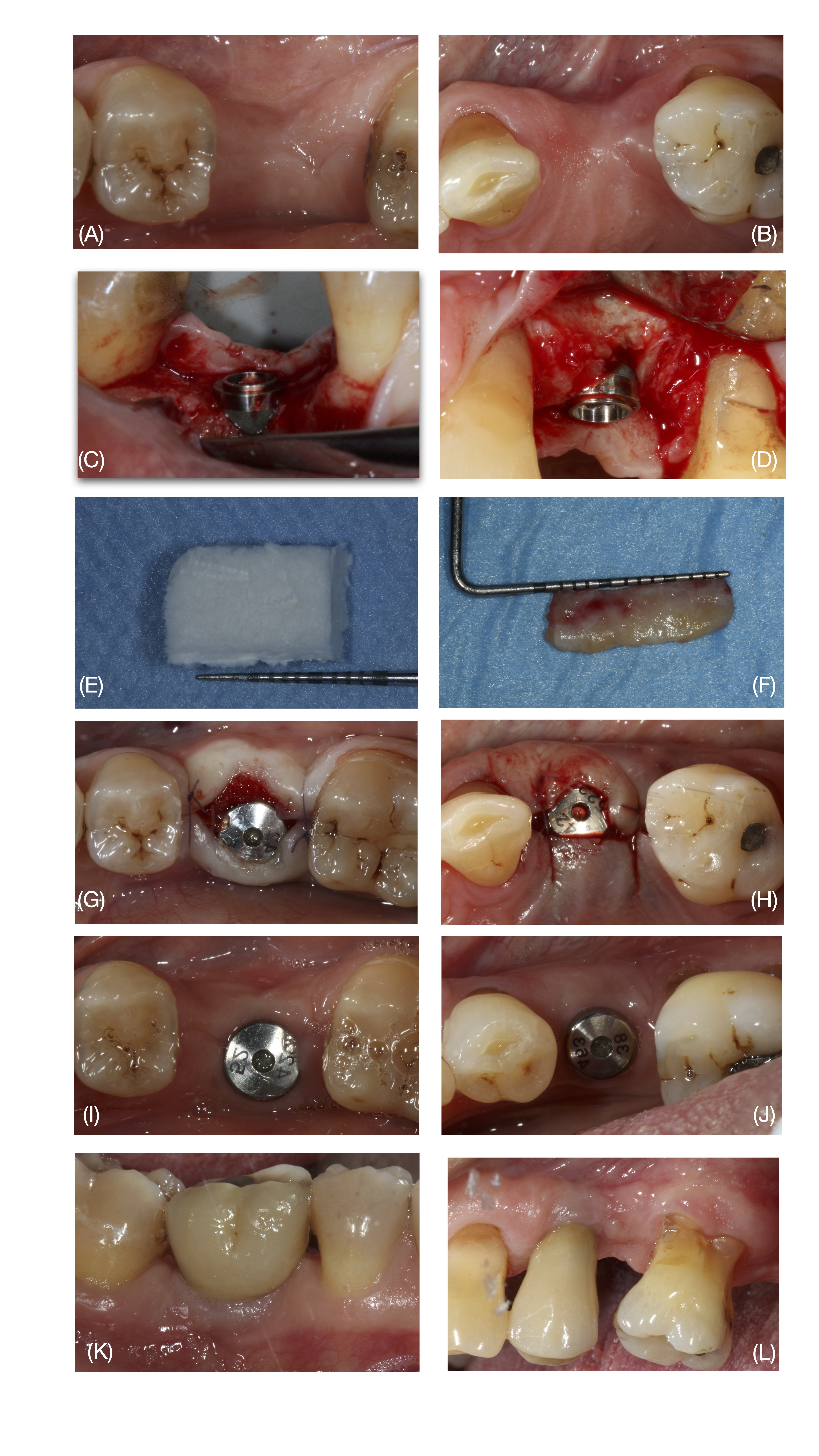

Supplement: Supplementary file 4 — Figure S3. Clinical procedures represented (left: VCMX, volume‐stable collagen matrix; right: CTG, de‐epithelized connective tissue graft). (A, B) Occlusal view displaying the volume deficiency. (C, D) Intrasurgical view of small buccal bone dehiscences at the implant positioning. (E, F) VCMX and CTG harvested from the palate after trimming. (G, H) Flap stabilization with sutures and wound closure. (I, J) Occlusal view at 3 months after surgery (3 M). Clinical pictures at 1 year after prosthetic loading (K, L). [file CLR-36-846-s005.tiff]

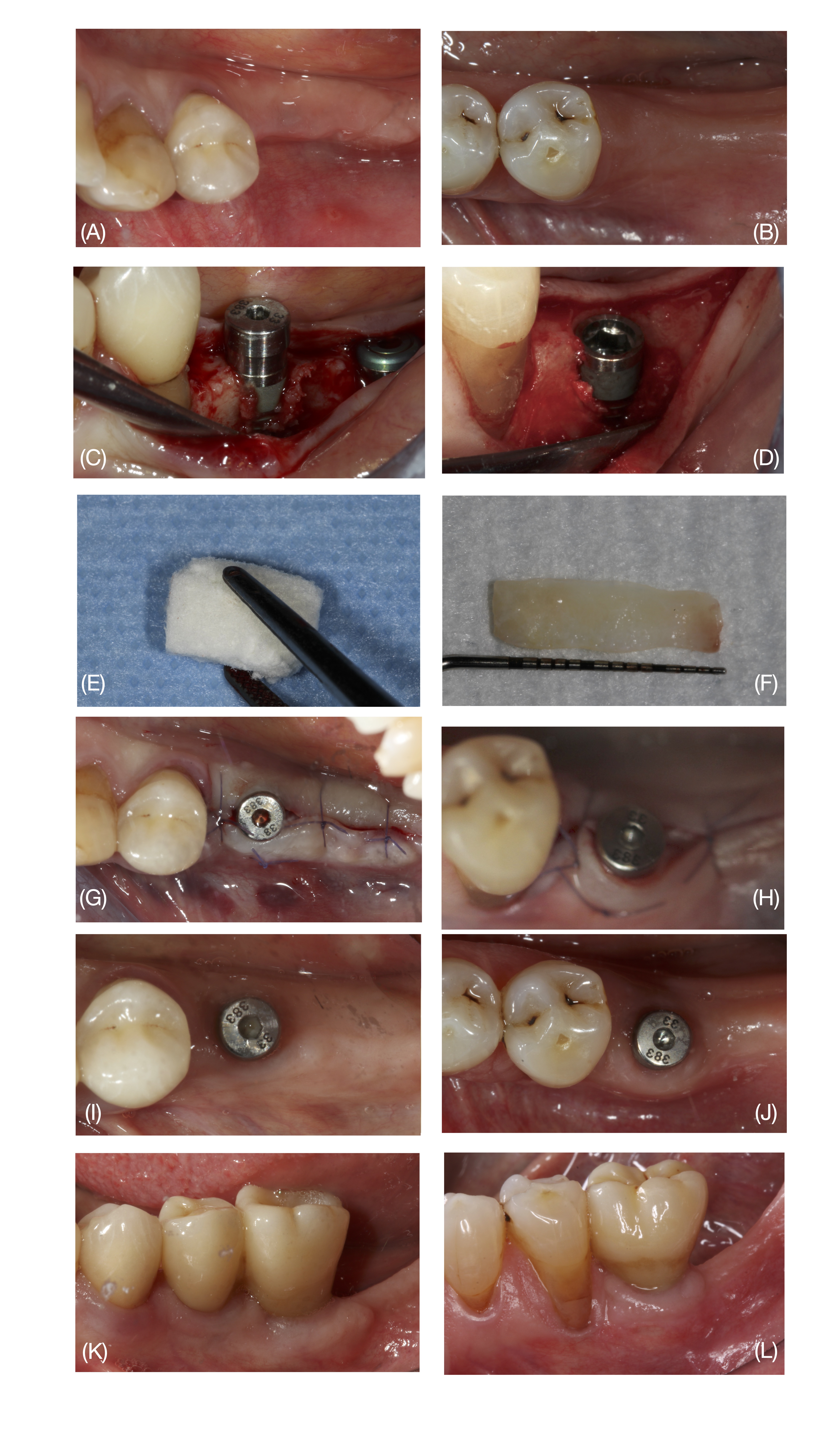

Supplement: Supplementary file 5 — Figure S4. Clinical procedures represented (left: VCMX, volume‐stable collagen matrix; right: CTG, de‐epithelized connective tissue graft). (A, B) Occlusal view displaying the volume deficiency. (C, D) Intrasurgical view of small buccal bone dehiscences at the implant positioning. (E, F) VCMX and CTG harvested from the palate after trimming. (G, H) Flap stabilization with sutures and wound closure. (I, J) Occlusal view at 3 months after surgery (3 M). Clinical pictures at 1 year after prosthetic loading (K, L). [file CLR-36-846-s002.tiff]
